# Happiness in Old Age: The Daughter Connection

**DOI:** 10.1007/s10902-023-00655-1

**Published:** 2023-04-28

**Authors:** Pataporn Sukontamarn, M. Niaz Asadullah, Nopphawan Photphisutthiphong, Yen Thi Hai Nguyen

**Affiliations:** 1 College of Population Studies, Chulalongkorn University, Bangkok, Thailand; 2 Faculty of Business and Economics, University of Malaya, Kuala Lumpur, Malaysia; 3 Monash University Malaysia, Selangor, Malaysia; 4 Vienna Institute of Demography, Austrian Academy of Sciences, Vienna, Austria

**Keywords:** Happiness, Older person, Ageing, Son bias, Thailand

## Abstract

Family and intergenerational relationships are becoming increasingly important as sources of support and care for the elderly population in rapidly ageing Asian societies. However, this has also raised concerns over reinforcement of cultural preference for sons as a source of old-age security. This paper therefore revisits the question—what determines happiness in old age—by investigating the role of adult children’s gender in the context of Thailand, an ageing Asian country with no legacy of sex-preference in fertility. We employ nationally representative data to examine the association between old-age happiness and presence of a co-residing child. Compared to living alone, living with at least one child is found to positively associate with older persons’ happiness. However, this result is specific to daughters. Moreover, compared to older men, women systematically benefit from a “daughter effect”. Co-residing daughters with university education and those who maintain a good relationship with their parents help explain the positive happiness effect on older persons. Co-residing daughters are also positively linked to  reduced loneliness; improved self-rated health; and improved economic conditions of older parents. Our findings suggest that policies that increase human capital of the girl child and enhance family solidarity are likely to have long term intergenerational wellbeing benefits.

## Introduction

Family and intergenerational relationships have become increasingly important as the populations of many countries are aging rapidly (Bengtson, 2018). In countries with insufficient institutional provisions for healthcare, the presence of adult children, living arrangements, and intergenerational support are crucial factors determining the subjective wellbeing of older persons (Grundy & Murphy, 2018; Teerawichitchainan et al., 2015). However, rapid aging also has implications for gender inequality, given the cultural preference for sons in many low-fertility Asian societies in the form of greater anticipated old-age support (Ebenstein & Leung, 2010; Silverstein et al., 2006a). Using data from Thailand, this paper, therefore, revisits the debate over the role of children as a source of happiness among parents in old age.

The available research on the subjective wellbeing of older persons has documented the role of a wide range of factors, including income, education, socioeconomic status, health, social engagement, active aging, and living arrangement (Angner et al., 2009; Hsu & Chang, 2015; Huang, 2019; Khodabakhsh, 2021; Matsuura & Ma, 2021; Ng et al., 2017; Ramia & Voicu, 2020; Srivastava & Muhammad, 2021; Utomo et al., 2019). While income is a predictor of old-age happiness, studies show that beyond a certain threshold value, the effect of income on happiness is often diminished. Instead, relational factors (e.g., time spent with friends, access to children, and the quality of the relationship) play a more prominent role in determining old-age happiness (Diener & Biswas-Diener, 2002).

The relationship between children and old-age happiness is, however, complex. On the one hand, children increase the happiness of older persons through financial, instrumental, and emotional support (Silverstein et al., 2006b; Tomassini et al., 2020). Studies have documented a positive association between support from children and the emotional wellbeing of older persons (Buber & Engelhardt, 2008; Silverstein & Bengtson, 1994; Zunzunegui et al., 2001). On the other hand, children may reduce the happiness of older persons if they feel that the children are a burden or a cause of worry to them (Li & Mutchler, 2020).

The link between old-age happiness and fertility is also contentious. Some studies find that older persons who are childless are more likely to be depressed (Zhang & Hayward, 2001). Moreover, if the number and the gender of the children do not meet the expectations of the parents, that could be detrimental to the subjective wellbeing of older persons (Shi, 2016). For example, in patriarchal societies, such as China and Vietnam, those who did not have a son were more likely to experience old-age depression (Chen & Silverstein, 2000). However, having many children can also be associated with reduced happiness. Studies have found that the number of children was negatively associated with the psychological wellbeing of older adults in certain settings, such as Myanmar and China (Tang et al., 2020; Teerawichitchainan et al., 2015; Williams et al., 2017).

While the existing research has also investigated how the gender of a coresiding child shapes old-age happiness, the relationship between the gender of children and old-age happiness is found to vary depending on the society’s cultural orientation (Teerawichitchainan et al., 2015; Tomassini et al., 2020). For example, in European countries, widows living with a child were happier than those living without a child; however, in Eastern and Southern Europe, it was only living with a daughter that the positive association was found (Grundy & Murphy, 2018). In contrast, in emerging Asian countries with a social norm of son preference, such as China, South Korea, and India, sons are valued as the main source of support for older parents, and the majority of older parents live with their sons (Das Gupta et al., 2003). In Vietnam, where son preference is well-documented, living with a married son is found to improve the emotional wellbeing of older persons (Teerawichitchainan et al., 2015).

Existing literatures have additionally documented the role of children in the provision of support for older parents. For developed countries, several studies have also documented the role of daughters (Raley & Bianchi, 2006; Silverstein et al., 2006b). For example, using the U.S. panel data, Silverstein et al. (2006b) found that parents’ increased need in terms of declining health was associated with more support from adult children. In particular, compared with sons, daughters provided more support to their mothers. Studies from Western countries have also found that daughters provided more emotional support than sons to their aging parents, especially to their mothers (Chesley & Poppie, 2009).

More recent studies suggest a changing role of daughters in old-age care in Asian societies (Cong & Silverstein, 2012; Ng et al., 2002; Yuan et al., 2021). In China and Taiwan, previous studies have found that sons were more likely to provide financial support to their older parents, while daughters were more likely to provide support in daily living (Cooney & Di, 1999; Lin et al., 2003; Sun, 2002; Zhan, 2004). However, recent research has found that in China, married daughters, especially coresiding ones, provided more financial support to parents compared with married sons (Xie & Zhu, 2009). Another study by Zeng et al. (2016) documented that having daughters was more beneficial than having sons in terms of the health outcome of older parents. Furthermore, recent studies have found that, even in patriarchal societies such as China, living with daughters is associated with greater happiness of older parents (Yuan et al., 2021), or that co-residence with sons as well as co-residence with daughters are beneficial to parents’ subjective wellbeing (Zhu et al., 2019).

Nonetheless, available research on the role of adult children’s gender on parental happiness in old age is complicated by the fact that in many Asian societies (e.g. China), there is a strong sex preference in fertility. This is often manifested in sex-selective abortion, resulting in the missing women phenomenon in the population and a daughter deficit (de Gara, 2017; Junhong, 2001; Miller, 2001; Rosenberg, 2009). The prenatal sex selection is thus the first step of possible selection bias, as sex-selective abortion arises from parents’ preference regarding the specific gender of their offspring. In particular, in a society where the number of children is restricted (e.g. due to the one-child policy in China earlier), the future co-residence between the older parents and a child of specific gender can be predetermined by the parents when making the decision regarding the girl fetus. This implies that existing empirical analysis (e.g. Zeng et al., 2016) of the link between children’s gender and parents’ wellbeing is likely to be biased.^1^ In contrast, the context of our study is Thailand, an aging Asian country with no legacy of sex selection in fertility (United Nations, 2019).

Thailand has experienced a demographic transition during the past six decades, with the old-age population accounting for 16.73% of the total population in 2019.^2^ At the same time, the country has witnessed changing patterns of living arrangements. The proportion of nuclear families declined in recent decades, while the proportion of extended families and single-person households increased (UNFPA, 2015). The Thai culture places importance on filial piety, and adult children play an important role in the support of aging parents (Croll, 2006; Teerawichitchainan et al., 2015). Studies have found that daughters contribute as much as, or more than, sons in the provision of support for older persons (Knodel et al., 1992). Therefore, compared with China and Vietnam, Thailand offers an interesting context in which one can reinvestigate the link between old-age happiness and children’s gender.

Our main research objective is to study the nexus between old-age happiness and the presence of a coresiding adult child in Thailand, with a focus on gender. More specifically, we answer the following questions: (i) What is the association between a coresiding child’s gender and old-age happiness? (ii) What characteristics of the child are associated with the happiness of older parents? (iii) What are the possible channels through which a coresiding child is associated with old-age happiness? The following three channels are investigated: improved health status, companionship, and economic security. To answer these research questions, we exploit data from a nationally representative household survey purposefully designed to study determinants of happiness among older persons in Thailand, with a focus on family structure and relationships. This enables us to estimate various multivariate econometric models isolating other factors that could potentially confound the influence of adult children’s gender on the happiness of elderly parents.

The rest of the paper is organized as follows. Section 2 describes the context of the study. Data, sample, and empirical methods are explained in Sect. 3, while the main results are presented in Sect. 4. Evidence on pathways underlying the main results is discussed in Sect. 5. Additional robustness tests are presented in Sect. 6. In Sect. 7, we conclude by briefly commenting on the policy significance of our results.

## Country Background and Study Context

**Fig. 1:**
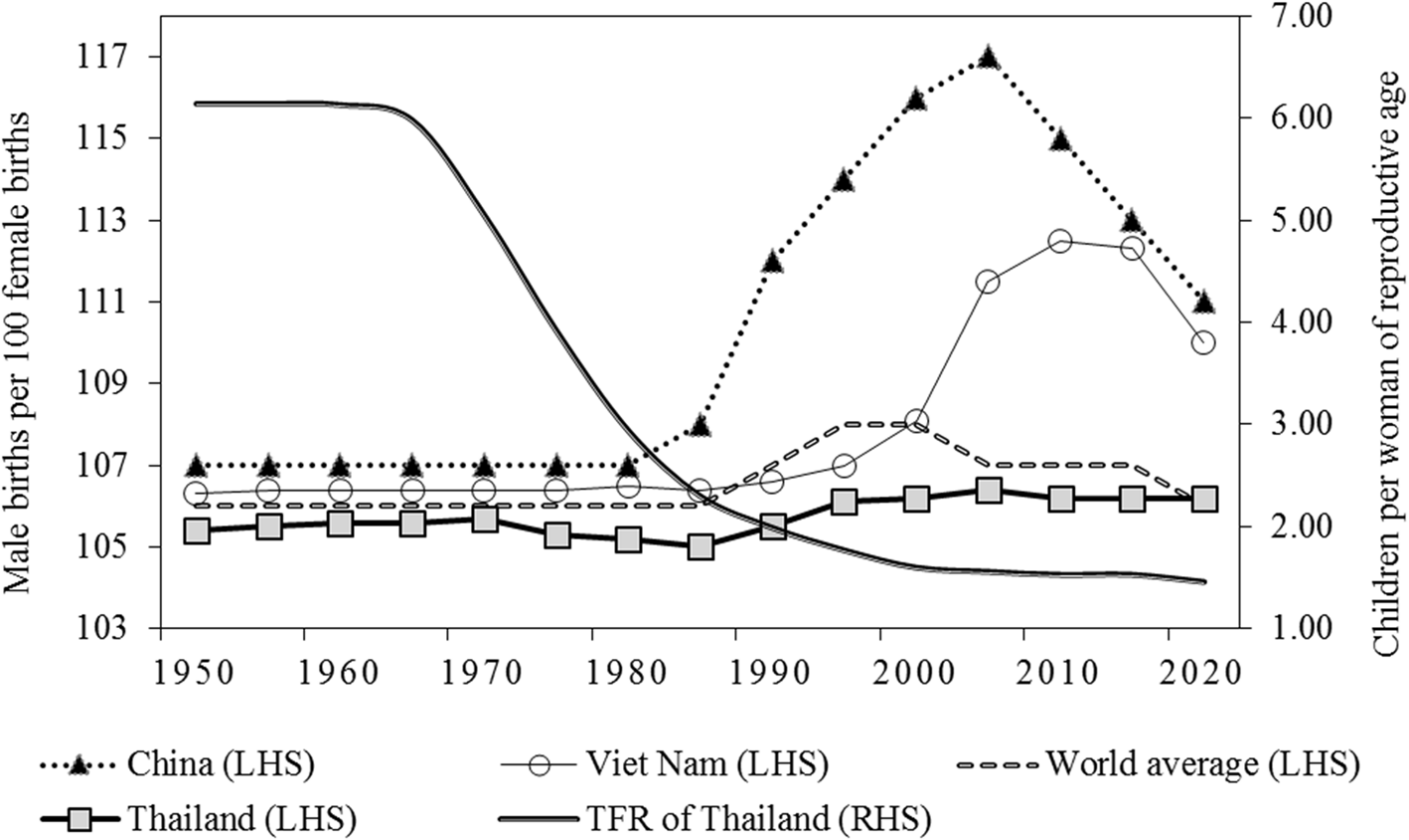
Total fertility rate in Thailand and comparison of sex ratios at birth in Thailand vis-à-vis China, Vietnam and the world average, 1950–2020. Sources: United Nations, Department of Economic and Social Affairs, Population Division (2019). *World Population Prospects 2019*, custom data acquired via website. Sex ratios at birth (as in male births per 100 female births) in Thailand, China, Vietnam and the world average are plotted on the primary vertical axis (LHS) and total fertility rate (as in children per woman of reproductive age) in Thailand is plotted on the secondary vertical axis (RHS)

Thailand has undergone a demographic transition in the past six decades, following a decline in TFR and a rise in life expectancy at birth. TFR in Thailand was 6.15 in 1960, which declined to 1.51 in 2019, while life expectancy at birth increased from 54.70 years in 1960 to 77.15 years in 2019 (World Bank, 2021a, b). In the post–Second World War period, high TFR was, in part, a result of the pronatalist government policy (Prachuabmoh & Mithranon, 2003). The first effort by the government to contain the population growth dates back to 1968 (Rosenfield et al., 1971). The Third National Economic and Social Development Plan (1972–1976) targeted a population growth rate of 2.5% by 1976. In this period, public health services supporting voluntary family planning, such as contraceptive use, IUD, and sterilization surgery, were provided in every province. Since then, TFR decreased substantially, as shown in Fig. 1. In addition, the delayed marriage of women contributed to the reduction of TFR (Knodel et al., 1996).

The change in the population structure significantly altered dependency ratios and economic support ratios.^3^ During the first demographic dividend witnessed in the early 1970s, there was a drastic fall in the child dependency ratio and a mild increase in the old-age dependency ratio. This implied a lesser economic burden on the working-age population and paid off in terms of the first demographic dividend.^4^ Afterward, the share of the elderly population continued to increase, while TFR remained low.

One notable feature of the country’s demographic transition is the absence of son bias in population statistics. Even with a declining TFR, there was no evidence of gender preference in Thailand. Unlike other Asian economies, such as China and Vietnam, where the number of male births exceeds the number of female births, the sex ratio at birth in Thailand has been steady and within the normal range. This has ensured a historically balanced proportion of daughters and sons in the population. Figure 1 compares the sex ratio at birth in three countries (i.e., China, Vietnam, and Thailand) with the world average. Before 1980, all three countries had the sex ratio at birth within the normal range. However, since the adoption of the one-child policy in 1980, the sex ratio at birth was on the rise in China. Vietnam too showed similar trends, with an increase in sex ratio at birth since 2005. This was partly due to prenatal sex determination, followed by selective abortion in Vietnam (Guilmoto et al., 2009). In contrast, as shown in Fig. 1, the sex ratio at birth in Thailand has been steady over time and in line with the world average.^5^

In 2019, the old-age population (11.13 million in total) accounted for 16.73% of the total population, and 44.18% of them were men.^6^ The transformation of Thailand into an aging society has created new challenges for the government and led to the adoption of a range of new policies. In 2003, the Act on the Elderly (2003 A.D.) was introduced to facilitate the implementation of policies on the protection and promotion of and support for older persons. In 2015, the Department of Older Persons was officially established under the Ministry of Social Development and Human Security, which introduced Universal Basic Income for older persons, an allowance scheme to alleviate poverty among the old-age population.^7^ Concerning healthcare, 83.2% of older persons were covered by the Universal Health Coverage scheme in receiving primary-care services.^8^ Alongside cash transfer schemes, institutional provisions for physical care have also improved. In 2015, there were 12 nursing homes under the Department of Older Persons, with a combined capacity to cater to 1200–1600 older persons (Department of Older Persons, n.d.). The private sector also plays a role in the caregiving service; there are over 100 registered firms that provide caregiving services to older persons.

Nonetheless, in practice, the above policy measures and provisions remain inadequate. Old-age care in Thailand still predominantly relies on informal care mechanisms. However, this has become a challenge, given the change in family structure, following the country’s demographic transition. According to the 2010 Population and Housing Census in Thailand,^9^ the average household size decreased from 5.2 persons per household in 1980 to 3.8 persons per household in 2000 and down to 3.1 persons per household in 2010. It is a social norm in Thailand for adult children to take care of their older parents when needed (Knodel et al., 2013). This may partly explain recent changes in family structure. While the proportion of nuclear families fell from 66.7% in 1987 to 49.9% in 2013, the proportion of extended families increased from 26.5 to 35.7% (UNFPA, 2015).^10^ It is these contrasting shifts in population structure and family arrangement that make Thailand, a low-fertility aging society characterized by an absence of son preference, ideal for our study on the link between old-age happiness and adult children’s gender. The next section elaborates on our data and research methodology.

## Methodology

### Data

This study utilizes data from the 2016 Population Change and Wellbeing in the Context of Ageing Society (PCWAS) Project. The project was administered by the College of Population Studies, Chulalongkorn University, together with four other universities in Thailand. The survey consisted of three instruments: (i) a household questionnaire, (ii) a questionnaire dedicated to women of reproductive age, and (iii) a questionnaire on individuals aged 60 and above. In terms of design, it was a multistage, stratified cluster sample survey. Overall, 15,222 households from 20 provinces in 5 regions (Bangkok, Northern region, Northeastern region, Central region, and Southern region) were available for interview. For each household, all older persons (aged 60 and above) were asked to answer the older persons’ questionnaire, resulting in a sample of 7450 older persons. Our study focuses on the happiness level of older persons, and this question was asked only to those who answered the questionnaire by themselves. Furthermore, observations with incomplete information were excluded. The final working sample comprised 6,129 older persons.

### Key Measurements

The main dependent variable “happiness level” represents the self-reported level of happiness measured on a Likert scale. The respondents were asked the following question: “From a score of 0–10, 0 means not happy at all, and 10 means very happy, how do you rate your level of happiness in the past 3 months?” The variable “happiness level” ranges from 0 to 10.

The main independent variables for the analysis are:

1. Living arrangements of older persons: This variable is defined as a categorical variable and represents four distinct types of living arrangements, indicating whether the respondent (i) lives with at least one child, (ii) lives with a spouse only (no coresident child), (iii) lives alone, or (iv) lives with other people who are neither the spouse nor children. For each case, living alone is the baseline category.
2. Gender of coresident children: The dummy variable “living with at least one child” is further disaggregated by the gender of coresident children in the following two ways:
  (i) Two groups: (a) only a coresident son(s) and (b) at least one coresident daughter
  (ii) Two groups: (a) only a coresident daughter(s) and (b) at least one coresident son
3. Characteristics of coresident daughters. The dummy variable “at least one coresident daughter(s)” is further disaggregated by the characteristic of a coresident daughter(s) in the following 3 ways: (i) marital status of a coresident daughter(s), (ii) relationship with a coresident daughter(s), and (iii) education of a coresident daughter(s).

### Empirical Model

For the main analysis, we follow Ferrer-i-Carbonell and Frijters (2004) and employ ordinary least squares (OLS) regressions to estimate the happiness equation. We focus on the gender of the coresident children, as children of different genders may take on different roles in the support and care of older parents. We then explore specific characteristics of the coresident children, namely, marital status, relationship with the older parents, and the level of education, to find out how these characteristics are associated with the happiness level of older parents. We perform separate regressions for older men and women, as coresidence with children as well as the children’s characteristics may influence their level of happiness differently. Formally, the main estimable equation is as follows:

1 $$Y_{i} = \alpha \, + {\mathbf{C}}_{{\mathbf{i}}} \beta \, + {\mathbf{X}}_{{\mathbf{i}}} {{\varvec{\uplambda}}} + {\mathbf{Z}}_{{\mathbf{i}}} \Omega \, + \, \varepsilon_{i}$$

where ***Y***_*i*_ represents the level of happiness of individual *i*; ***C***_***i***_ is a vector reflecting coresidence with children (e.g. whether the respondent lives with at least one child, whether there is at least one coresident daughter or whether there are only coresident sons); ***X***_***i***_ is a vector reflecting living arrangements in the case that there is no coresident child (e.g. whether the respondent lives with a spouse only); ***Z***_***i***_ represents a vector of individual characteristics (e.g. gender, age, years of education, marital status, religion, whether the respondent has at least one child, and the number of children who are still alive). Individual characteristics vector ***Z***_***i***_ also includes economic indicators (e.g. work status and personal income). A health indicator is represented by self-rated health and consists of four groups: (i) very good, (ii) good, (iii) normal, and (iv) bad or very bad. We also control for the place of residence (urban/rural) and the region of residence. *ε*_*i*_ is the error term

For coresidence with children (i.e. vector ***C***_***i***_), we estimate three versions of Eq. (1). For Model 1, the main variable of interest is whether the respondent lives with at least one child. To explore the role of different genders, Model 2 focuses on whether there is at least one coresident daughter or whether there are only coresident sons. Model 3 employs variables that indicate whether there is at least one coresident son or whether there are only coresident daughters.

In terms of living arrangements (i.e. vector ***X***_***i***_), for older persons with no coresident child, we control for whether the respondent lives with a spouse only or belongs to other types of living arrangements (the reference group is living alone). For Model 1, if there is no coresident child, we control for whether at least one child is living next door. If not, we control for whether at least one child is living in the same village. For Models 2 and 3, if there is no coresident child, we control for whether at least one daughter is living next door. If not, we control for whether at least one son is living next door. If not, then we control for whether at least one daughter is living in the same village. If not, then we control for whether at least one son is living in the same village.

For further investigation, we test for specific pathways through which coresidence with children may be associated with the level of happiness of older persons. Previous works of literature have demonstrated that support from children, especially coresident children, benefits older parents in terms of physical health, mental health, and financial status (Chesley & Poppie, 2009; Cong & Silverstein, 2012; Xie & Zhu, 2009; Zeng et al., 2016; Zhan, 2004).^11^ Based on data availability, we test for the following channels: (i) health status (whether self-rated health was normal, good, or very good in the past week), (ii) loneliness and worry (whether the respondent felt lonely/worried sometimes or often in the past month), and (iii) perceived sufficiency of income (whether income was adequate/more than adequate). For each of the three channels, the dependent variable takes on the value of 0 or 1, and Probit regression analysis is employed. The independent variables are the same as in the main analysis.

Lastly, our analysis assumes that there is no concern of selection effect. It is possible that there are unobserved factors that may influence both happiness and the decision to coreside with daughters or sons. Regardless of the child’s gender, an additional selection bias relates to co-residency status in general. Older parents with high unobserved support needs are more likely to coreside with at least one child (Manacorda & Moretti, 2006) and/or a child of particular gender. To address the second type of bias, some causal studies have estimated instrumental variables and endogenous treatment effect model (Do & Malhotra, 2012; Yuan et al., 2021). These issues are not considered in the present research and as such, our estimates should not be interpreted as causal.

## Summary Statistics

Table 1 presents the summary statistics of the study variables. The average level of happiness of our sample was 7.88 out of 10. There was almost no difference between the average level of happiness of older males and older females (7.89 and 7.88, respectively). A higher proportion of older males reported that their self-rated health was good compared with older females (0.81 and 0.72, respectively). Loneliness and worry were more common among older females compared with older males. Slightly over half of older males (0.56) and older females (0.55) reported that their income was sufficient. Concerning living arrangements, over half of older persons in the sample lived with at least one child. Concerning living arrangements by the gender of children, 0.31 of older males and 0.33 of older females lived with at least one daughter. A slightly lower proportion lived with at least one son (0.29 for both older males and older females). Around 0.12 of older males and 0.11 of older females had no coresident child but had at least one child living next door. The average age of older males and females in the sample was around 69. On average, older males had 5.99 years of education, while older females had 4.96 years. In terms of marital status, 0.83 of older males and only 0.50 of older females were married. Around 0.96 of older males and 0.91 of older females had at least one child. On average, older males and females had around three children (3.08).

**Table 1:** Summary statistics

Variables	All		Males		Females	
	Mean/proportion	s.d	Mean/proportion	s.d	Mean/proportion	s.d
*Main dependent variable*						
Happiness level (range: 0–10)	7.88	1.96	7.89	1.89	7.88	2.00
*Other dependent variables*						
Self-rated health: fine*	0.76	0.43	0.81	0.39	0.72	0.45
Loneliness*	0.29	0.45	0.25	0.43	0.31	0.46
Worry*	0.43	0.50	0.37	0.48	0.47	0.50
Having sufficient income*	0.56	0.50	0.56	0.50	0.55	0.50
*Main independent variables*						
*Living arrangement*						
Living with at least one child*	0.54	0.50	0.53	0.50	0.55	0.50
Living with spouse only*	0.27	0.44	0.35	0.48	0.21	0.40
Living alone*	0.11	0.31	0.08	0.27	0.12	0.33
Other types of living arrangement*	0.08	0.28	0.04	0.18	0.12	0.32
*Living arrangement by gender of children*						
Living with son(s) only*	0.22	0.41	0.22	0.41	0.22	0.41
Living with daughter(s) only*	0.25	0.43	0.24	0.43	0.26	0.44
Living with both son(s) and daughter(s)*	0.07	0.26	0.07	0.26	0.07	0.26
Living with at least one daughter*	0.32	0.47	0.31	0.46	0.33	0.47
Living with at least one son*	0.29	0.45	0.29	0.46	0.29	0.45
*Living arrangement by characteristics of co-resident daughter(s)*						
At least one married co-resident daughter*	0.19	0.39	0.17	0.38	0.20	0.40
Only non-married co-resident daughter(s)*	0.14	0.34	0.14	0.35	0.14	0.34
At least one co-resident daughter with good relationship*	0.29	0.46	0.28	0.45	0.30	0.46
Only co-resident daughter(s) with normal or bad relationship*	0.03	0.17	0.03	0.17	0.03	0.17
At least one co-resident daughter with university education*	0.13	0.33	0.13	0.34	0.13	0.33
Only co-resident daughter(s) with secondary education or lower*	0.20	0.40	0.18	0.39	0.21	0.41
*Other control variables*						
*Proximity of nearest child (no co-resident child)*						
Nearest child: next-door*	0.11	0.32	0.12	0.32	0.11	0.31
Nearest child: same village*	0.06	0.24	0.06	0.24	0.06	0.23
*Proximity of nearest child by gender*						
Nearest child: next-door & is a daughter*	0.07	0.26	0.07	0.26	0.07	0.26
Nearest child: next-door & is a son (no daughter next-door)*	0.04	0.20	0.04	0.20	0.04	0.19
Nearest child: same village & is a daughter*	0.04	0.19	0.04	0.20	0.03	0.18
Nearest child: same village & is a son (no daughter in the same village)*	0.02	0.15	0.02	0.15	0.02	0.15
*Personal characteristics*						
Age (range: 60–99)	68.81	7.00	68.62	6.79	68.94	7.14
Female*	0.59	0.49	0	0	1.00	0
Years of education	5.39	4.15	5.99	4.32	4.96	3.96
Married*	0.64	0.48	0.83	0.37	0.50	0.50
Single*	0.04	0.20	0.01	0.12	0.06	0.25
Widowed*	0.27	0.45	0.12	0.33	0.38	0.49
Divorced/separated*	0.05	0.21	0.03	0.17	0.06	0.23
Buddhist*	0.96	0.20	0.96	0.20	0.96	0.20
Non-Buddhist*	0.04	0.20	0.04	0.20	0.04	0.20
Has at least one child	0.93	0.26	0.96	0.20	0.91	0.29
Number of children who are still alive (range: 0–13)	3.08	1.97	3.10	1.88	3.07	2.03
*Economic indicators*						
Worked in the past week*	0.46	0.50	0.56	0.50	0.39	0.49
Did not work in the past week*	0.54	0.50	0.44	0.50	0.61	0.49
Personal income below 40,000 baht per year*	0.51	0.50	0.46	0.50	0.54	0.50
Personal income between 40,000 and 99,999 baht per year*	0.25	0.43	0.26	0.44	0.24	0.43
Personal income between 100,000 and 299,999 baht per year*	0.17	0.37	0.19	0.39	0.15	0.36
Personal income 300,000 baht per year or higher*	0.08	0.27	0.09	0.29	0.07	0.25
*Health indicator*						
Self-rated health is very good*	0.05	0.22	0.06	0.24	0.04	0.20
Self-rated health is good*	0.30	0.46	0.34	0.47	0.27	0.44
Self-rated health is normal*	0.40	0.49	0.40	0.49	0.41	0.49
Self-rated health is bad or very bad*	0.24	0.43	0.19	0.39	0.28	0.45
*Place of residence*						
Urban*	0.46	0.50	0.42	0.49	0.48	0.50
Rural*	0.54	0.50	0.58	0.49	0.52	0.50
*Region of residence*						
Bangkok*	0.12	0.33	0.11	0.32	0.13	0.34
North*	0.22	0.42	0.23	0.42	0.22	0.41
Northeast*	0.25	0.43	0.26	0.44	0.24	0.43
Central*	0.20	0.40	0.19	0.39	0.21	0.40
South*	0.20	0.40	0.20	0.40	0.21	0.40
N	6129		2539		3590	

“Happiness level” is self-reported level of happiness in the past 3 months from the score of 0 to 10, where 0 means not happy at all and 10 means very happy. * denotes a dummy variable which takes on the value 1 for the stated characteristic, and 0 otherwise

## Main Results

Table 2 presents the main results of the study (i.e., estimates of Eq. 1). We first present estimates for the full sample. Gender-specific regression estimates are presented in Table 3. We find that compared with living alone, living with at least one child is positively associated with older persons’ happiness (see column 1, Table 2). Columns 1 and 2 of Table 3 show that the positive association between having a coresiding child and old-age happiness holds in the case of older females but not in the case of older males. We further explore the role of the coresiding children’s gender. The results together suggest that the positive association between having a coresiding child and older persons’ happiness is specific to daughters. Column 2 of Table 2 and columns 3 and 4 of Table 3 divide the variable “living with at least one child” into two groups: (i) only a coresident son(s) and (ii) at least one coresident daughter. Column 3 of Table 2 and columns 5 and 6 of Table 3 divide the variable “living with at least one child” into (i) only a coresident daughter(s) and (ii) at least one coresident son. The findings together confirm that living with at least one daughter is positively associated with the happiness of older persons, in particular older females.

**Table 2:** OLS regression estimates of Happiness equation (main correlate of interest: co-residence with children): All older persons

	Happiness level of older persons					
	(1)		(2)		(3)	
	Coef	(t-stats)	Coef	(t-stats)	Coef	(t-stats)
Living with at least one child	0.192	(1.99)**				
Only co-resident son(s)			− 0.003	(0.03)		
Only co-resident daughter(s)					0.337	(3.29)***
At least one co-resident daughter			0.328	(3.28)***		
At least one co-resident son					0.060	(0.60)
Living with spouse only	0.125	(1.21)	0.120	(1.16)	0.119	(1.15)
Other types of living arrangement	− 0.032	(0.27)	− 0.032	(0.28)	− 0.034	(0.29)
Nearest child: next-door	0.112	(1.24)				
Nearest child: same village	− 0.027	(0.24)				
Nearest child: next-door & is a daughter			0.116	(1.10)	0.113	(1.07)
Nearest child: next-door & is a son			0.122	(0.93)	0.121	(0.93)
Nearest child: same village & is a daughter			− 0.110	(0.80)	− 0.112	(0.81)
Nearest child: same village & is a son			0.121	(0.72)	0.120	(0.72)
Age	0.135	(2.32)**	0.138	(2.37)**	0.137	(2.37)**
Age squared	− 0.001	(2.37)**	− 0.001	(2.43)**	− 0.001	(2.44)**
Female	0.204	(3.85)***	0.203	(3.83)***	0.201	(3.79)***
Years of education	0.003	(0.38)	0.003	(0.39)	0.003	(0.40)
Marital status: Single	− 0.209	(1.12)	− 0.214	(1.15)	− 0.216	(1.16)
Marital status: Widowed	− 0.005	(0.08)	− 0.012	(0.16)	− 0.010	(0.15)
Marital status: Divorced or separated	− 0.317	(2.55)**	− 0.315	(2.54)**	− 0.320	(2.59)***
Non-Buddhist	0.014	(0.12)	0.016	(0.13)	0.032	(0.27)
Whether the respondent has at least one child	− 0.106	(0.71)	− 0.099	(0.66)	− 0.113	(0.76)
Has at least one child*number of children	− 0.028	(1.84)*	− 0.032	(2.08)**	− 0.026	(1.67)*
Worked in the past week	0.114	(2.11)**	0.119	(2.21)**	0.116	(2.16)**
Personal income: 40,000–99,999 baht per year	0.193	(3.18)***	0.197	(3.26)***	0.192	(3.18)***
Personal income: 100,000–299,999 baht per year	0.396	(5.37)***	0.397	(5.39)***	0.398	(5.41)***
Personal income: 300,000 baht per year or higher	0.302	(2.73)***	0.297	(2.69)***	0.301	(2.72)***
Self-rated health: very good	1.663	(14.06)***	1.671	(14.16)***	1.663	(14.08)***
Self-rate health: good	1.200	(17.87)***	1.189	(17.74)***	1.192	(17.77)***
Self-rate health: normal	0.548	(8.89)***	0.544	(8.84)***	0.546	(8.86)***
Area of residence: urban	0.048	(0.89)	0.046	(0.86)	0.051	(0.94)
Constant	1.914	(0.92)	1.823	(0.88)	1.872	(0.90)
N	6129		6129		6129	
R-squared	0.10		0.10		0.10	

Happiness score ranges from 0 to 10. Absolute values of t statistics are in parentheses.

**Significant at 5%; *** significant at 1%. The baseline group for marital status is “married”. The baseline group for personal income is “income below 40,000 baht per year”. The baseline group for self-rated health is “bad or very bad”. The baseline group for area of residence is “rural”. All regressions control for region of residence

**Table 3:** OLS regression estimates of Happiness equation (main correlate of interest: co-residence with children): Older males and older females

	Happiness level of older persons					
	Males	Females	Males	Females	Males	Females
	(1)	(2)	(3)	(4)	(5)	(6)
Living with at least one child	−0.024	0.278				
	(0.15)	(2.24)**				
Only co-resident son(s)			− 0.265	0.108		
			(1.56)	(0.80)		
Only co-resident daughter(s)					0.119	0.425
					(0.71)	(3.22)***
At least one co-resident daughter			0.124	0.402		
			(0.76)	(3.11)***		
At least one co-resident son					− 0.164	0.148
					(0.99)	(1.13)
Living with spouse only	− 0.135	0.256	− 0.155	0.257	− 0.150	0.255
	(0.83)	(1.83)*	(0.95)	(1.84)*	(0.92)	(1.83)*
Other types of living arrangement	− 0.305	0.075	− 0.318	0.077	− 0.319	0.075
	(1.32)	(0.54)	(1.38)	(0.56)	(1.38)	(0.54)
Nearest child: next-door	0.088	0.135				
	(0.67)	(1.08)				
Nearest child: same village	− 0.034	− 0.014				
	(0.21)	(0.09)				
Nearest child: next-door & is a daughter			0.155	0.102	0.153	0.099
			(1.02)	(0.71)	(1.00)	(0.69)
Nearest child: next-door & is a son			− 0.010	0.212	− 0.010	0.211
			(0.05)	(1.18)	(0.06)	(1.17)
Nearest child: same village & is a daughter			− 0.075	− 0.155	− 0.077	− 0.157
			(0.39)	(0.80)	(0.40)	(0.81)
Nearest child: same village & is a son			0.056	0.206	0.055	0.206
			(0.23)	(0.90)	(0.23)	(0.90)
Control variables	Yes	Yes	Yes	Yes	Yes	Yes
N	2539	3590	2539	3590	2539	3590
R-squared	0.12	0.09	0.13	0.09	0.13	0.09

Happiness score ranges from 0 to 10. Absolute values of t statistics are in parentheses

**Significant at 5%; *** significant at 1%. Control variables are the same as those listed in Table 2

The results in Table 3 show that, for older females, living with a spouse only is positively associated with the level of happiness, compared with living alone. The results in Tables 2 and 3 show that, for older persons with no coresident child, having children living next door or in the same village is not significantly associated with the level of happiness, compared with having children living further away.

The results in Table 2 suggest that several demographic and socioeconomic characteristics are associated with the level of happiness of older persons. Controlling for other factors, older females are happier than older males. The results show an inverted U-shaped relationship between age and happiness. Happiness initially increases from the age of 60 to around 68 and then declines afterward. Older persons who are divorced or separated are less happy than those who are married. Income is positively associated with happiness. As expected, health is also positively associated with happiness. Work is associated with a higher level of happiness in older persons.

Overall, the findings in Tables 2 and 3 suggest that living with at least one daughter is positively associated with the happiness of older persons, in particular older females. However, living with at least one son is not significantly associated with the happiness of older persons.

Table 4 further explores the characteristics of coresident daughters, which are associated with the happiness level of older persons. The three characteristics investigated are marital status, relationship with the older person, and education level. For all columns, the control variables are the same as those in Table 2. Columns 1 and 2 include the interaction term between “having at least one coresident daughter” and “at least one coresident daughter is married.” The results suggest that, for older females, irrespective of the marital status of the coresident daughter(s), having at least one coresident daughter is positively associated with the level of happiness.

**Table 4:** OLS regression of Happiness equation (controlling for characteristics of daughters)

	Happiness level of older persons					
	Males	Females	Males	Females	Males	Females
	(1)	(2)	(3)	(4)	(5)	(6)
At least one co-resident daughter	0.097	0.374	− 0.447	− 0.227	0.131	0.246
	(0.54)	(2.56)**	(1.77)*	(1.02)	(0.76)	(1.80)*
At least one co-resident daughter*At least one married	0.051	0.046				
daughter	(0.39)	(0.40)				
At least one co-resident daughter*At least one daughter with			0.632	0.694		
good relationship			(2.98)***	(3.48)***		
At least one co-resident daughter*At least one daughter with					− 0.018	0.407
university education or higher					(0.13)	(3.42)***
Only co-resident son(s)	− 0.265	0.107	− 0.268	0.111	− 0.266	0.109
	(1.56)	(0.80)	(1.58)	(0.83)	(1.56)	(0.81)
Living with spouse only	− 0.155	0.257	− 0.157	0.267	− 0.155	0.272
	(0.95)	(1.84)*	(0.96)	(1.91)*	(0.95)	(1.95)*
Other types of living arrangement	− 0.318	0.077	− 0.319	0.079	− 0.318	0.079
	(1.38)	(0.56)	(1.38)	(0.57)	(1.38)	(0.57)
Control variables	Yes	Yes	Yes	Yes	Yes	Yes
N	2539	3590	2539	3590	2539	3590
R-squared	0.13	0.09	0.13	0.09	0.13	0.09

Happiness score ranges from 0 to 10. Absolute values of t statistics are in parentheses

**Significant at 5%; *** significant at 1%. Control variables are as listed in Table 2. For proximity of children, we use the variables indicating proximity of children by gender of children

Columns 3 and 4 show that the interaction term between “having at least one coresident daughter” and “at least one coresident daughter has a good/very good relationship with the older person” is positively and statistically significant for both columns. On the other hand, the level term “having at least one coresident daughter” is negatively and statistically significant in column 3. For older males, compared with living alone, living with only daughters with normal or bad relationships is negatively associated with the level of happiness. However, compared with living alone, living with at least one daughter with whom they have a good or very good relationship is positively associated with the level of happiness. For older females, compared with living alone, living with only daughters with normal or bad relationships is not significantly associated with the level of happiness, while living with at least one daughter with whom they have a good or very good relationship is positively associated with the level of happiness.

Columns 5 and 6 investigate the level of education of a coresident daughter(s). The interaction term between “having at least one coresident daughter” and “at least one coresident daughter has a university or higher education” is positively and statistically significant in column 6. In addition, the level term “having at least one coresident daughter” is positively and statistically significant in column 6 only. For older females, compared with living alone, living with only a daughter(s) with upper secondary education or lower is positively associated with the level of happiness. Furthermore, living with at least one daughter with a university education or higher is positively associated with the level of happiness of older females compared with living with one with upper secondary education or lower.

Taken together, the results in Table 4 suggest that living with at least one daughter with whom the older person has a good relationship is positively associated with the happiness level of both older males and females. For older females, while living with at least one daughter with any level of education is positively associated with happiness compared with living alone, living with at least one daughter with a university education or higher further increases the magnitude of the association between living with at least one daughter and the level of happiness.

## Additional Results and Pathways

Given the systematic link between happiness in old age and adult children’s gender as documented in Sect. 5, we test for specific pathways through which the observed correlation could arise in our data. In total, four possible channels are considered: (i) the improvement in self-rated health, (ii) the reduction of feeling of loneliness, (iii) the reduction of worry, and (iv) the sufficiency of income. The corresponding binary dependent variables are (i) whether self-rated health in the past month, (ii) whether the respondent felt lonely sometimes or often in the past month, (iii) whether the respondent felt worried sometimes or often in the past month, and (iv) whether income was adequate or more than adequate. Probit regression analysis is employed. The results are reported in the Appendix (Tables 6, 7, 8, 9).

Overall, the results suggest that living with at least one daughter is positively associated with the probability of having good health for older females. For loneliness, regardless of the gender of the child, living with at least one child is negatively associated with the probability of feeling lonely for both older males and females. Moreover, living with a daughter with whom they have a good relationship or a daughter with a university education or higher is associated with a lower probability of feeling worried in older women. For both older men and women, compared to living alone, living with a daughter with a university education or higher is positively associated with good financial conditions as captured through perceived adequacy of income.

## Robustness Tests

In this section, we revisit the stability of our results to three methodological concerns: (a) a linear specification of the happiness function, (b) the use of a full sample versus restricting the sample to only those with children, and (c) missing happiness score for a subsample of respondents. As mentioned earlier, our main estimates are based on OLS models, since Ferrer-i-Carbonell and Frijters (2004) show that modeling happiness scores as only cardinal (as is done in OLS regressions) or ordinal (as is done in ordered latent response models) makes little practical difference. Moreover, our data rejects the parallel lines assumption required for the valid estimation of ordered logit models. Nonetheless, we have also conducted a logistic regression analysis by redefining our dependent variable to be 1 if the respondent reported having a level of happiness between 8 and 10 and 0 if the respondent reported having a level of happiness between 0 and 7 (the logistic regression estimates are not reported but are available upon request). As the mean of the happiness level is 7.88, those reporting a happiness level of 8 or higher can be considered to be “above the mean” in terms of happiness level (referred to as “happy”), and those reporting a happiness level of 7 or lower can be considered to be “below the mean” (referred to as “not so happy”). The logistic results are very similar to the OLS results presented in the paper. For older women, living with at least one daughter is associated with a higher probability of being “happy.” For both older men and older women, living with at least one daughter with whom the older person has a good relationship is associated with a higher probability of being “happy.” Furthermore, for older women, living with at least one daughter with a university education or higher is associated with a higher probability of being “happy.” However, as our happiness measure ranges from 0 to 10, we employed linear regression specification to fully explore the variation in the level of happiness (as presented in Tables 2, 3, 4).

For the second methodological concern, intergenerational research on the elderly population faces the challenge of whether to include all elderly respondents in the analysis or to include only those with children. For the results presented in Tables 2, 3, 4, we have also conducted the same analysis using the sample of older persons with at least one living child, as suggested by a referee. The results are very similar to the results based on the sample of all older persons (the results are available upon request). Given no significant change in results based on the conditional sample, we have used the sample of all older persons in the main results section. The justification for this is that, by doing so, we avoid concerns related to sample selection bias (since having children is a matter of choice and, for that reason, the probability of observing an elderly person conditional on having children is nonrandom).

The third methodological concern relates to item nonresponse. In earlier studies based on survey data of older persons, Koyama et al. (2014) and Kutschar et al. (2019) show that the increase in item nonresponse was associated with (older) age and the level of cognitive ability of nursing home residents. Empirical analysis that ignores item nonresponse rate can be potentially biased unless the characteristics of the included and the excluded observations are balanced (Lindeboom et al., 2002; Thomas et al., 2013). Our data also shows that the respondents’ characteristics in the working sample (N = 6129) vis-a-vis the excluded group (N = 1321) are significantly different in several sociodemographic dimensions, such as age, health conditions, economic status, presence of children, gender, and years of education.

One possible explanation for the differences is that the dependent variable “happiness level” came from the question that was asked only to the older persons who answered the questionnaire by themselves. The question was not asked to those who had another person helping them to answer the questions in the interview process. Therefore, those who had other people helping them to answer the questionnaire were excluded. Those who had other people helping them during the interview were more likely to be old and in bad health. To deal with missing values of observation in the sample, a few methods have been suggested in the literature, such as (i) removal of incomplete observations and analysis of the complete cases (our current approach); (ii) imputation method in which the statistically simulated values are replaced for the missing values (Nishimura et al., 2016); (iii) sample selection model (Heckman, 1976). An earlier study by Koné et al. (2019) applied the Heckman selection model to eliminate the problem of selection bias arising from nonresponse items and obtain unbiased estimates of parameters.

To test whether our results specific to the “daughter effect” are driven by sample selection bias, we implemented the Heckman correction. Two variables—“having at least one ADL difficulty” and “number of household members”—were included additionally in the selection (first) stage. These two variables were hypothesized to predict the probability of reporting a happiness score (e.g., elderly respondents with at least one ADL difficulty are less likely to report their subjective wellbeing) but not affect the level of happiness. Both variables are found to be significant in the selection stage. Reassuringly, in the outcome equation, the lambda term is significant and negative, implying that factors that lead to nonmissing data on happiness are likely to be associated with lower happiness scores. Yet, this correction did not alter our main results specific to the “daughter effect” (see Table 5). We have repeated the Heckman model for all other specifications, and the conclusion remains unchanged (additional estimates of the Heckman model are not reported here but are available upon request).

**Table 5:** Main equation Heckman selection model estimates of happiness equation (main correlate of interest: co-residence with children)

	Happiness level of older persons (0–10)								
	All	Males	Females	All	Males	Females	All	Males	Females
	(1)	(2)	(3)	(4)	(5)	(6)	(7)	(8)	(9)
Living with at least one child	0.400	0.346	0.385						
	(3.83)***	(2.02)**	(2.91)***						
Only co-resident son(s)				0.173	0.117	0.160			
				(1.56)	(0.65)	(1.13)			
Only co-resident daughter(s)							0.564	0.478	0.566
							(5.08)***	(2.67)***	(3.98)***
At least one co-resident daughter				0.573	0.521	0.552			
				(5.21)***	(2.92)***	(3.94)***			
At least one co-resident son							0.254	0.214	0.225
							(2.34)**	(1.20)	(1.63)
Living with spouse only	0.340	0.343	0.336	0.220	0.395	0.217	0.401	0.205	0.397
	(3.37)***	(3.39)***	(3.34)***	(1.36)	(2.99)***	(1.34)	(3.04)***	(1.27)	(3.02)***
Other types of living arrangement	0.078	0.081	0.074	− 0.339	0.193	− 0.350	0.198	− 0.363	0.193
	(0.62)	(0.65)	(0.59)	(1.35)	(1.30)	(1.39)	(1.33)	(1.45)	(1.30)
Control variables	Yes	Yes	Yes	Yes	Yes	Yes	Yes	Yes	Yes
Total number of observations	7450	3119	4331	7450	3119	4331	7450	3119	4331
Selected	6129	2539	3590	6129	2539	3590	6129	2539	3590
Non-selected	1321	580	741	1321	580	741	1321	580	741
Lambda (Inverse Mills Ratio)	− 1.172	− 1.067	− 1.152	− 1.234	− 1.15	− 1.201	− 1.177	− 1.062	− 1.172
	(4.88)***	(3.28)***	(3.36)***	(5.15)***	(3.55)***	(3.51)***	(4.91)***	(3.27)***	(3.43)***

Absolute values of z statistics are in parentheses.

**Significant at 5%; *** significant at 1%. Control variables are similar to those listed in Table 2, however, the following independent variables were missing for a number of observations, and were not included in the Heckman correction models: education, marital status, work status, income, self-rated health, and the relationship with co-resident daughters

## Conclusion

In this paper, we revisit the literature on happiness among elderly persons in rapidly aging emerging economies, where despite economic modernization, traditional family-centric support provisions remain critical for the wellbeing of older persons. We have achieved this by utilizing data from a survey designed and implemented by the members of the study team in Thailand, a country with a balanced population sex ratio and no legacy of son preference in fertility.

Altogether, the results show that compared with living alone, living with at least one adult child is positively and significantly associated with older persons’ happiness. In particular, older females benefit most from living with at least one child. However, among this subsample, it is living with at least one daughter that is positively associated with happiness in old age; living with at least one son does not show a significant association with older parents’ happiness. Further investigation into the characteristics of coresident daughters shows that the relationship between the daughter and the older person and the level of education of the daughters are the two drivers of older persons’ happiness. Living with at least one daughter with whom the older person has a good relationship is positively associated with the happiness level of both older males and females. For older females, living with daughters with a university education or higher is positively associated with the level of happiness.

Living with daughters is associated with happiness through four channels: (i) an improvement in self-rated health (for older females), (ii) a reduction in loneliness (for both older males and females), (iii) a reduction in emotional ill-being or worry (for older females, in the case of daughters with a good relationship with their parents or a university education or higher), and (iv) improvement in economic conditions in terms of income sufficiency (for both older males and females, in the case of daughters with a university education or higher).

This study adds to the burgeoning literature on the role of children in the subjective wellbeing of older persons by highlighting the connection between living with at least one daughter and old-age happiness (Chen & Short, 2008; Chesley & Poppie, 2009; Silverstein et al., 2006b; Yuan et al., 2021). Our findings are also aligned with the emerging evidence on China, where despite the social norm of son preference, recent research (e.g. Yi et al., 2016) found that older parents were happier as a result of the gratitude of and the good relationship with their daughters than their sons. Our results that living with daughters is associated with improved self-rated health of older persons are in line with the findings that having daughters was more beneficial than having sons in terms of the health outcomes for Chinese older parents (Zeng et al., 2016). Moreover, our findings that living with daughters with a university education or higher is associated with improved economic conditions also agree with recent findings from China that daughters provided more financial support to parents compared with sons (Hu, 2017; Xie & Zhu, 2009). On the other hand, the “daughter effect” on older parents’ happiness presented here contrast with findings from Vietnam, a country with the social norm of son preference, where living with a married son was found to improve the emotional wellbeing of older persons while living with a daughter did not (Teerawichitchainan et al., 2015).

Nonetheless, our study has some limitations. First, the interplay of life satisfaction between generations is not considered due to the lack of available data. So far, the discussions have mainly considered the role of adult children on the older parents’ subjective wellbeing. On the other hand, the life satisfaction of the older parents can also be transmitted to adult children. Such studies can be accomplished by using longitudinal data. The life satisfaction of older parents, particularly the mothers, influences the life satisfaction of adult children; this can be explained by the maternal bond during the child-rearing period (Dobewall et al., 2019; Headey et al., 2014). Another limitation of the study is that we have not delved into the reason for the living arrangements of the older persons, in particular the decision to coreside with their children. From the perspective of the children, there exists a reciprocal benefit of living with their older parents that can potentially explain the decision of adult children to coreside with their parents. It is also the desire of adult children, especially those who do not have their own house, to live with their parents to receive accommodation, financial assistance, and childcare support from the older parents (Li & Wu, 2019; Zhang et al., 2014). In addition, it is possible that there are unobserved factors, such as the willingness or the ability of a child to take care of the older parents or the availability of a child to live with the parents, which influence both the decision to coreside with daughters or sons and happiness level. This may lead to potential selection bias. If so, our results are not causal as we have also cautioned in Sect. 3.3.

The limitations aside, our findings have important policy implications for other rapidly aging emerging Asian economies. The results suggest that children’s human capital can have added social returns in terms of improvements in the subjective and emotional wellbeing of the elderly population. Therefore, policies that increase investment in female schooling are likely to have long-term effects on the subjective wellbeing of older persons and ensure that the benefits of the second and the third demographic dividends are fully reaped.

## Appendix

See Tables 6, 7, 8, 9.

**Table 6:** Channels through which daughters matter for parental happiness: Self-rated health (1 = self-rated health is fine, 0 = self-rated health is bad)

	Males	Females	Males	Females	Males	Females	Males	Females	Males	Females
	(1)	(2)	(3)	(4)	(5)	(6)	(7)	(8)	(9)	(10)
Living with at least one child	0.049	0.062								
	(1.46)	(2.17)**								
Living with at least one daughter			0.047	0.075	0.067	0.057	0.013	0.006	0.044	0.081
			(1.39)	(2.56)**	(1.83)*	(1.72)*	(0.24)	(0.13)	(1.24)	(2.61)***
At least one co-resident daughter*At least					− 0.041	0.030				
one married daughter					(1.35)	(1.14)				
At least one co-resident daughter*At least							0.039	0.076		
one daughter with good relationship							(0.91)	(1.73)*		
At least one co-resident daughter*At least									0.008	− 0.017
one daughter with university education or higher									(0.27)	(0.59)
Only co-resident son(s)			0.047	0.042	0.046	0.041	0.047	0.042	0.047	0.042
			(1.36)	(1.37)	(1.34)	(1.36)	(1.35)	(1.39)	(1.36)	(1.37)
Control variables	Yes	Yes	Yes	Yes	Yes	Yes	Yes	Yes	Yes	Yes
Pseudo R-squared	0.04	0.04	0.04	0.04	0.04	0.04	0.04	0.04	0.04	0.04
N	2539	3590	2539	3590	2539	3590	2539	3590	2539	3590

Absolute values of z statistics are in parentheses. ** significant at 5%; *** significant at 1%. Marginal effects are reported. Control variables are as listed in Table 2, excluding the level of self-rated health (which came from the same question as the dependent variable in this Table). For proximity of children, columns (1) and (2) use the variables indicating proximity of children regardless of gender, while columns (3)–(10) use the variables indicating proximity of children by gender of children

**Table 7:** Channels through which daughters matter for parental happiness: Loneliness (1 = feeling lonely sometimes or often, 0 = not feeling lonely)

	Males	Females	Males	Females	Males	Females	Males	Females	Males	Females
	(1)	(2)	(3)	(4)	(5)	(6)	(7)	(8)	(9)	(10)
Living with at least one child	− 0.133	− 0.149								
	(3.65)***	(5.08)***								
Living with at least one daughter			− 0.161	− 0.168	− 0.172	− 0.173	− 0.125	− 0.114	− 0.157	− 0.144
			(4.68)***	(5.78)***	(4.50)***	(5.17)***	(2.23)**	(2.26)**	(4.28)***	(4.63)***
At least one co-resident daughter*At least					0.025	0.009				
one married daughter					(0.71)	(0.33)				
At least one co-resident daughter*At least							− 0.046	− 0.065		
one daughter with good relationship							(0.91)	(1.41)		
At least one co-resident daughter*At least									− 0.012	− 0.075
one daughter with university education or higher									(0.35)	(2.54)**
Only co-resident son(s)			− 0.065	− 0.103	− 0.064	− 0.103	− 0.064	− 0.103	− 0.065	− 0.103
			(1.74)*	(3.43)***	(1.73)*	(3.44)***	(1.74)*	(3.44)***	(1.75)*	(3.42)***
Control variables	Yes	Yes	Yes	Yes	Yes	Yes	Yes	Yes	Yes	Yes
Pseudo R-squared	0.09	0.07	0.09	0.07	0.09	0.07	0.09	0.07	0.09	0.08
N	2539	3590	2539	3590	2539	3590	2539	3590	2539	3590

Absolute values of z statistics are in parentheses. ** significant at 5%; *** significant at 1%. Marginal effects are reported. Control variables as listed in Table 2. For proximity of children, columns (1) and (2) use the variables indicating proximity of children regardless of gender, while columns (3)–(10) use the variables indicating proximity of children by gender of children

**Table 8:** Channels through which daughters matter for parental happiness: Worry (1 = feeling worried sometimes or often, 0 = not feeling worried)

	Males	Females	Males	Females	Males	Females	Males	Females	Males	Females
	(1)	(2)	(3)	(4)	(5)	(6)	(7)	(8)	(9)	(10)
Living with at least one child	0.117	0.000								
	(2.65)***	(0.01)								
Living with at least one daughter			0.095	− 0.015	0.084	− 0.013	0.175	0.089	0.134	0.006
			(2.03)**	(0.43)	(1.67)*	(0.33)	(2.51)**	(1.49)	(2.73)***	(0.16)
At least one co-resident daughter*At least					0.019	− 0.003				
one married daughter					(0.52)	(0.11)				
At least one co-resident daughter*At least							− 0.085	− 0.114		
one daughter with good relationship							(1.54)	(2.14)**		
At least one co-resident daughter*At least									− 0.094	− 0.055
one daughter with university education or higher									(2.60)***	(1.73)*
Only co-resident son(s)			0.156	0.023	0.156	0.023	0.156	0.022	0.155	0.022
			(3.18)***	(0.63)	(3.18)***	(0.63)	(3.19)***	(0.61)	(3.16)***	(0.62)
Control variables	Yes	Yes	Yes	Yes	Yes	Yes	Yes	Yes	Yes	Yes
Pseudo R-squared	0.05	0.06	0.05	0.06	0.05	0.06	0.05	0.06	0.05	0.06
N	2539	3590	2539	3590	2539	3590	2539	3590	2539	3590

Absolute values of z statistics are in parentheses. ** significant at 5%; *** significant at 1%. Marginal effects are reported. Control variables are as listed in Table 2. For proximity of children, columns (1) and (2) use the variables indicating proximity of children regardless of gender, while columns (3)–(10) use the variables indicating proximity of children by gender of children

**Table 9:** Channels through which daughters matter for parental happiness: Income sufficiency (1 = sufficient income, 0 = insufficient income)

	Males	Females	Males	Females	Males	Females	Males	Females	Males	Females
	(1)	(2)	(3)	(4)	(5)	(6)	(7)	(8)	(9)	(10)
Living with at least one child	− 0.089	− 0.042								
	(1.90)*	(1.24)								
Living with at least one daughter			− 0.065	− 0.016	− 0.059	− 0.017	− 0.094	− 0.088	− 0.108	− 0.081
			(1.33)	(0.45)	(1.11)	(0.42)	(1.26)	(1.48)	(2.12)**	(2.18)**
At least one co-resident daughter*At least					− 0.010	0.001				
one married daughter					(0.26)	(0.03)				
At least one co-resident daughter*At least							0.031	0.079		
one daughter with good relationship							(0.51)	(1.50)		
At least one co-resident daughter*At least									0.116	0.176
one daughter with university education or higher									(2.98)***	(5.58)***
Only co-resident son(s)			− 0.137	− 0.077	− 0.137	− 0.077	− 0.137	− 0.076	− 0.134	− 0.076
			(2.68)***	(2.10)**	(2.69)***	(2.10)**	(2.69)***	(2.09)**	(2.64)***	(2.07)**
Control variables	Yes	Yes	Yes	Yes	Yes	Yes	Yes	Yes	Yes	Yes
Pseudo R-squared	0.12	0.10	0.12	0.10	0.12	0.10	0.12	0.10	0.12	0.11
N	2539	3590	2539	3590	2539	3590	2539	3590	2539	3590

Absolute values of z statistics are in parentheses. ** significant at 5%; *** significant at 1%. Marginal effects are reported. Control variables are as listed in Table 2. For proximity of children, columns (1) and (2) use the variables indicating proximity of children regardless of gender, while columns (3)–(10) use the variables indicating proximity of children by gender of children

## Footnotes

1 Based on our literature search, we didn’t find any study that methodologically corrected for sex-selective abortion related biases and how that affects research on children’s gender and parental happiness in China. The only two studies that corrected for possible selection bias—Yuan et al. (2021) and Zhu et al. (2019)—limit the focus to inter-generational co-residence status.

2 The Bureau of Registration Administration (BORA), Ministry of Interior, *Official statistics registration database*.

3 The dependency ratio includes child dependency ratio (number of children aged 0–14 per 100 working-age population) and old-age dependency ratio (number of older persons aged 65 or older per 100 working-age population). The economic support ratio is defined as the ratio of the number of effective workers to the number of effective consumers in the National Transfer Accounts (NTA) (Mason et al., 2017).

4 At the macro level, this dividend was estimated to account for 15.5% of economic growth (Mason & Kinugasa, 2008).

5 According to the World Health Organization, the natural sex ratio at birth to balance the number of male and female population in the life course is between 102 and 106 males per 100 females. This is because men are likely to die earlier than women (Case & Paxson, 2005).

6 The Bureau of Registration Administration (BORA), Ministry of Interior, *Official statistics registration database*.

7 At present, an older person receives his/her old-age allowance on the age-related basis, i.e., the monthly income of 600 baht (19.17 USD) for those in their 60 s, 700 baht (22.36 USD) for those in their 70 s, 800 baht (25.56 USD) for those in their 80 s, and 1000 baht (31.95 USD) for those in their 90 s or older. The Bank of Thailand reference rate of baht against U.S. dollar in 2020 is 31.30 baht per USD.

8 Other schemes are the civil servants’ medical benefit scheme under the Ministry of Finance and the social security scheme under the Ministry of Labor. Some older persons also had their own private health insurance.

9 The 2020 Population and Housing Census in Thailand was postponed due to the COVID-19 pandemic.

10 The proportion of single-person households also rose from 6.1 to 13.9%.

11 From the theoretical perspective, the life domain approach to explaining happiness, which is based on the bottom-up model, states that happiness can be viewed as the net outcome of reported satisfaction with various domains of life (Campbell et al., 1976; Easterlin, 2006). Satisfaction in each domain is the product of both objective conditions and aspirations in that domain (Easterlin, 2006). The domains of life include economic condition, family life, health, and work (Easterlin and Sawanfa, 2007).
